# Copper sulfide nanoparticles as high-performance cathode materials for Mg-ion batteries

**DOI:** 10.1038/s41598-019-43639-z

**Published:** 2019-05-29

**Authors:** Kostiantyn V. Kravchyk, Roland Widmer, Rolf Erni, Romain J.-C. Dubey, Frank Krumeich, Maksym V. Kovalenko, Maryna I. Bodnarchuk

**Affiliations:** 1 Laboratory of Inorganic Chemistry, Department of Chemistry and Applied Biosciences, ETH Zürich, Vladimir-Prelog-Weg 1, CH-8093 Zürich, Switzerland; 2 Laboratory for Thin Films and Photovoltaics, Empa – Swiss Federal Laboratories for Materials Science and Technology, Überlandstrasse 129, CH-8600 Dübendorf, Switzerland; 3 Nanotech@surfaces Laboratory, Empa – Swiss Federal Laboratories for Materials Science and Technology, Überlandstrasse 129, CH-8600 Dübendorf, Switzerland; 4 Electron Microscopy Center, Empa – Swiss Federal Laboratories for Materials Science and Technology, Überlandstrasse 129, CH-8600 Dübendorf, Switzerland

**Keywords:** Energy, Nanoparticles

## Abstract

Rechargeable magnesium batteries are appealing as safe, low-cost systems with high-energy-density storage that employ predominantly dendrite-free magnesium metal as the anode. While significant progress has been achieved with magnesium electrolytes in recent years, the further development of Mg-ion batteries, however, is inherently limited by the lack of suitable cathode materials, mainly due to the slow diffusion of high-charge-density Mg-ions in the intercalation-type host structures and kinetic limitations of conversion-type cathodes that often causes poor cyclic stability. Nanostructuring the cathode materials offers an effective means of mitigating these challenges, due to the reduced diffusion length and higher surface areas. In this context, we present the highly reversible insertion of Mg-ions into nanostructured conversion-type CuS cathode, delivering high capacities of 300 mAh g^−1^ at room temperature and high cyclic stability over 200 cycles at a current density of 0.1 A g^−1^ with a high coulombic efficiency of 99.9%. These materials clearly outperform bulk CuS, which is electrochemically active only at an elevated temperature of 50 °C. Our results not only point to the important role of nanomaterials in the enhancement of the kinetics of conversion reactions but also suggest that nanostructuring should be used as an integral tool in the exploration of new cathodes for multivalent, i.e., (Mg, Ca, Al)-ion batteries.

## Introduction

Rechargeable Mg-ion batteries are considered an attractive energy storage system for both mobile and stationary energy storage applications^1^. Unlike metallic Li, Na or K, metallic Mg foils can be used as anodes due to the predominantly^2^ smooth, fast and dendrite-free electrodeposition of Mg, and reduced fire hazards related to this metal. Metallic Mg anodes also present numerous other advantages, such as the high natural abundance of Mg and its largely explored reserves, its high volumetric (3833 mAh cm^−3^) and gravimetric (2205 mAh g^−1^) capacities, high safety and suitably low electrode potential (−2.4 V vs SHE)^3–5^. Some of the challenges facing Mg-ion batteries are as follows. With respect to the anode, highly stable magnesium oxide (MgO) can readily form from traces of moisture or some oxygen-containing solvents/electrolytes. Thin films of MgO can fully deactivate the Mg anode, as this oxide is neither electronically nor ionically conductive to a sufficient degree. This is one of the reasons for using highly reductive electrolyte formulations. Hence, the major bottleneck in the development of Mg-ion batteries has been the lack of suitable electrolytes that can support reversible plating of Mg on the anode, yet provide a suitable voltage range for operating the cathode. In recent years, this voltage range had expanded from less than 2 V to ca. 3.5 V^6–10^. This progress stimulates and enables the exploration of novel cathode materials. In this field, there are important differences between Mg^2+^ and monovalent Li^+^ and Na^+^ ions. Intercalation of Mg^2+^ ions, which are as small as Li-ions but have a much higher charge density, is found to be sluggish in most cases, due to strong coulombic interactions with the negatively charged host lattice^11^. A closely related problem is the desolvation of Mg-ions at the electrolyte-cathode interface, where strong coordination of these ions prevents rapid insertion into the cathode or fully hampers this process^12^. Nearly all known magnesium cathode materials are characterized by their limited rate-capabilities and cyclabilities, as well as their high overpotentials between charging and discharging, yielding low energy efficiency^1,4,13–20^. The chevrel phase (Mo_6_S_8_) developed by Aurbach *et al*.^21^ in the 2000s remains the best known Mg-ion cathode. Its theoretical energy density is estimated to be *ca*. 126 Wh kg^−1^ based on the reported operation voltage of ~1.2 V and specific charge-storage capacity of 110 mAh g^−1^. Cathode materials offering higher operational voltages and higher capacities are thus urgently needed in order to narrow the gap between Mg-ion batteries and Li-ion batteries, where the latter exhibit theoretical energy densities are 360–450 Wh kg^−1^, due to their higher voltages of 2.8–4.4 V and cathodic capacities are 130–300 mAh g^−1^ ^22–26^.

To date, creating nanostructures in Mg-ion-storing cathode materials has been the only plausible strategy for improving the overall kinetics of reversible Mg^2+^ insertion by drastically decreasing the in-solid diffusion path (Figure S1)^11,27–33^. Nanostructuring has been somewhat controversial in the field of Li(Na)-ion batteries for both anode and cathode materials: besides presenting obvious new opportunities (broader scope of usable electrode materials), it has exhibited important shortcomings, the foremost of which is due to enhanced formation of the solid-electrolyte interface (SEI) due to the higher surface area^34–38^. This SEI layer is often soft and highly unstable. Formation of SEI consumes Li(Na)-ions and leads to irreversible capacity loss. In contrast to Li(Na)-ion batteries, SEI formation issues are different for Mg-ion batteries. Mg electrolytes are usually stable at the potentials needed for Mg electroplating/stripping, and hence do not lead to the formation of SEI on the anode side (Mg foil)^3,5^. However, similarly to Li/Na cathodes, Mg cathodes with the high surface area have enhanced side reactions with the electrolyte, which might lead to SEI formation. On the other hand, the sluggish Mg diffusivity in Mg cathodes makes nanostructuring the most, and essentially only, effective general strategy for ensuring a satisfactory degree of magnesiation over practically relevant time-scales and temperatures. Bulk cathodes often allow magnesiation at elevated temperatures. Some recent examples of nanostructured Mg cathode materials include TiS_2_ nanotubes^27^, MoS_2_ nanoribbons^28^, VO_*x*_ nanotubes^29^, and nano-sized, open-frame, conformable V_2_O_5_^30^, all of which exhibited higher capacities, energy efficiencies and rate capabilities compared to their bulk counterparts.

In this work, we were motivated to probe nanostructuring approaches in order to study conversion-type copper (II) sulfide cathodes for Mg-ion batteries. CuS offers one of the highest available capacities at 560 mAh g^−1^ and has a high electrical conductivity of 10^3^ S cm^−1^ ^39–47^. The CuS conversion electrodes reported to date, however, suffered from reduced rate capabilities and cycling stabilities at room temperature, which is associated with the large structural reconstruction of the electrodes during cycling^44^. This leads to large volume changes and thus destruction of the electrodes. Specifically, up to recently, the best cycling stability tests for CuS cathodes at room temperature showed a rather low gravimetric capacity of 153 mAh g^−1^ after 20 cycles, with a low capacity retention of 75% and a large voltage hysteresis, resulting in a poor energy efficiency of 68%^46^. Notably, Fei Xu *et al*.^48^ reported on CuS NPs as cathodes for Mg-ion batteries, using commercial 90–150 nm CuS NPs and achieved a high charge-storage capacity of 175 mAh g^−1^ at a current density of 50 mA g^−1^. In this context, nanostructuring and the formulation of suitable soft composites could be an effective way to accommodate large structural changes in conversion-type cathode materials and enhance the kinetics of the electrochemical conversation reactions.

Herein, we report that nanostructured CuS in the form of 20 nm nanoparticles (NPs) delivers at room temperature a high capacity of 300 mAh g^−1^ at a current density of 100 mA g^−1^, outperforming its bulk counterparts. Bulk CuS showed negligible electrochemical activity at room temperature, which improved only at an elevated temperature of 50 °C. The mechanism of magnesiation/demagnesiation in CuS NPs has been probed by X-ray photoelectron spectroscopy (XPS) and energy-dispersive X-ray spectroscopy (EDX).

## Results and Discussion

**Figure 1:**
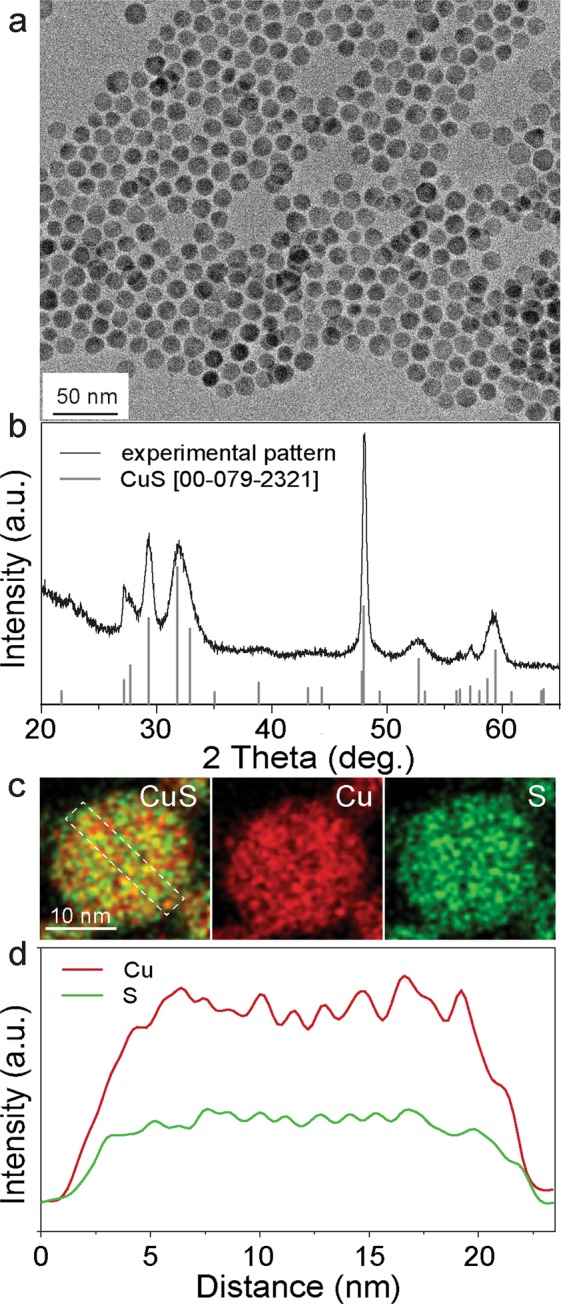
TEM image (**a**) and XRD pattern (**b**) of CuS NPs. X-ray wavelength is λ = 1,54056 Å. The reflection at 2 Theta of 48 degrees ((110) peak) was narrower and sharper in comparison with other reflections suggesting that CuS NPs were grown along directions perpendicular to the c-axis and have platelet shape morphology^49^. The elemental maps of a single CuS NP using EDX measurements in the HAADF-STEM mode for Cu + S, Cu and S (**c**); the white frame shows the area from where the line scans were extracted. (**d**) Line scans representing the intensity distribution of Cu (red) and S (green) within CuS NPs.

In short, monodisperse CuS NPs were synthesized using a previously reported method by heating up of copper (I) chloride, oleylamine (Oam), oleic acid (OA) and octadecene to 180 °C, following the injection of 1 M solution of sulfur in oleylamine^49^. Transmission electron microscopy (TEM) and X-ray diffraction (XRD) (Fig. 1a,b and S2) confirmed the formation of uniform and highly crystalline hexagonal CuS NPs (space group *P63/mmc*, *a* = *b* = 3.788 Å, *c* = 16.333 Å, JCPDS No. 00-079-2321) with sizes on the order of 20 nm. EDX measurements of CuS NPs in high-angle annular dark-field scanning transmission electron microscopy mode (HAADF-STEM) revealed that Cu and S were uniformly distributed throughout each NP (Fig. 1c,d). After the synthesis, highly insulating long-chain capping ligands (OA/Oam) were removed through mild chemical treatment using a hydrazine-based ligand-stripping protocol that was initially developed for colloidal quantum dots (see Figure S3)^50^. For the electrochemical measurements, the CuS electrodes were prepared by mixing a powder of the CuS NPs with carbon black (CB), polyvinylidene fluoride (PVdF) and N-methylpyrrolidone (NMP). The resulting slurries were cast onto a tungsten current collector. Coin-type cells were employed for the electrochemical tests. The cell consisted of magnesium foil as the counter and reference electrode, CuS as the working electrode, and a glass-fiber separator that was placed between both electrodes and soaked with magnesium electrolytes based on Mg(HMDS)_2_/AlCl_3_/MgCl_2_ in tetraglyme^51,52^.

**Figure 2:**
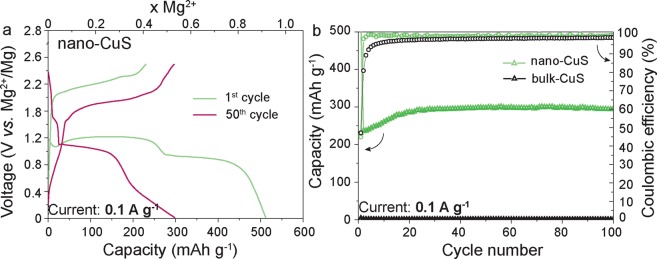
Electrochemical performance of CuS NPs. (**a**) Galvanostatic charge-discharge curves during the 1^st^ and 50^th^ cycle at a current density of 0.1 A g;^−1^ (**b**) Cyclic stability measured at a current density of 0.1 A g^−1^.

Figure 2a shows the voltage profiles of the Mg ion battery at a current density of 0.1 A g^−1^ using CuS NPs as the cathode material. During the first discharge, two distinct plateaus at approximately 1.2 V and 0.9 V vs. Mg^2+^/Mg were observed, indicating a multi-step Mg insertion and reaction mechanism. The insertion of approximately 0.48 and 0.43 moles (first and second plateaus) of Mg^2+^ ions per mole of CuS corresponds to the total initial discharge capacity of 512 mAh g^−1^. Only about half of the Mg cations can be extracted on the first charge because the others are trapped in the structure. From the second cycle onward, the reversibility improved dramatically, and the capacity stabilized at approximately 300 mAh g^−1^ with a high coulombic efficiency of 99.9% (Fig. 2b). Relatively high capacities of 110–212 mAh g^−1^ were measured for CuS NPs even at high current densities varied from, accordingly, 1 to 0.2 A g^−1^ (Figure S4).

**Figure 3:**
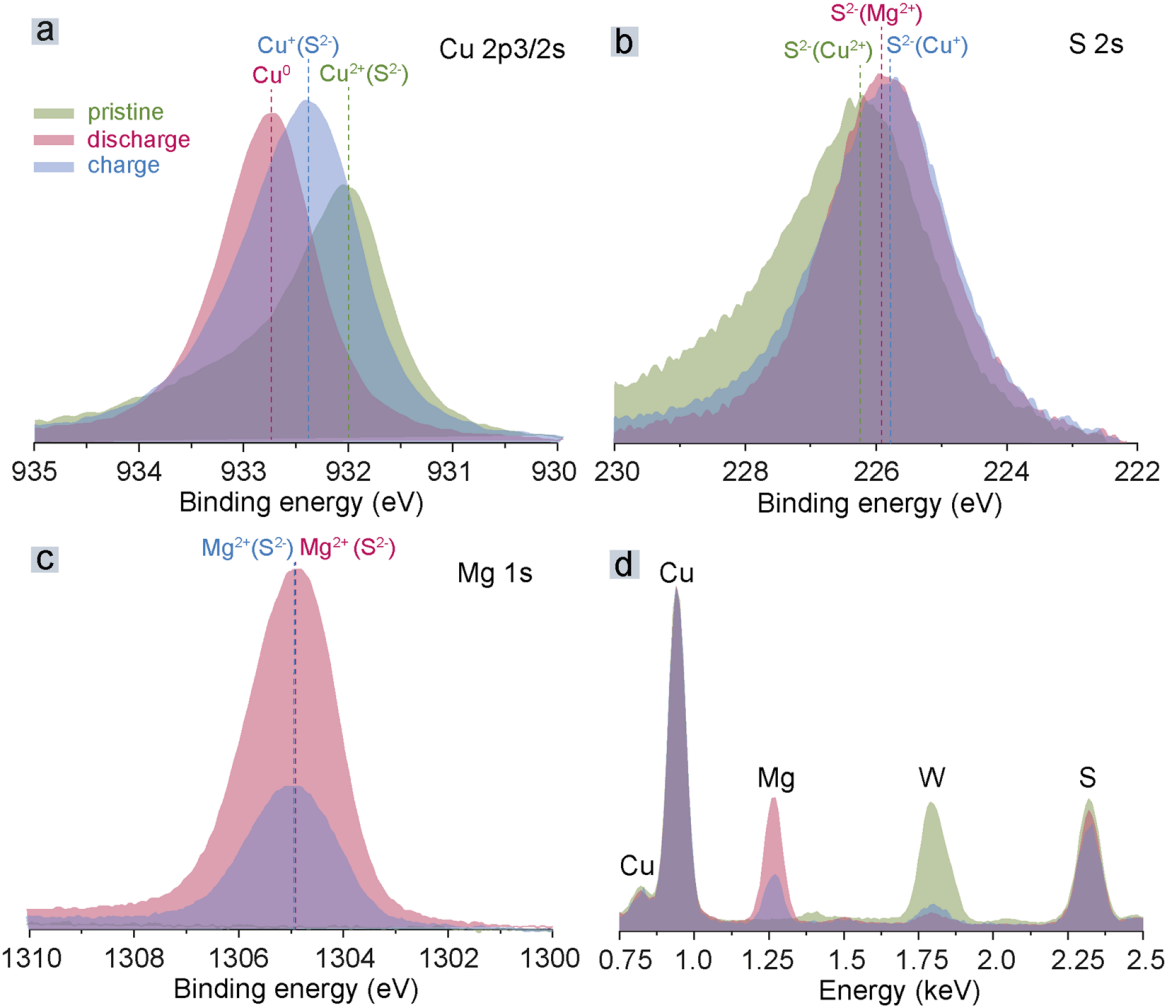
*Ex situ* XPS (**a**,**b**,**c**) and EDX (**d**) measurements of electrodes composed of CuS NPs after discharge and charge. Atomic ratios of S, Cu and Mg for pristine, discharged and charged CuS NPs derived from corresponding XPS spectra are shown in the Table S1. The intensities of EDX spectra were normalized to the intensity of Cu peaks. Prior to these measurements, the electrodes were rinsed from the Mg electrolyte with pure tetraglyme.

The detailed Mg^2+^ insertion mechanism was analyzed by *ex situ* XPS and EDX methods with the pristine, discharged and charged electrodes. Figure 3a–c show the changes in the Cu 2p_3/2_, S 2 s and Mg 1 s XPS peaks. After discharge, the Cu 2p_3/2_ peak position shifted towards a higher binding energy, indicating the reduction of Cu^2+^ towards the formation of metallic copper. After the first charge, copper is only oxidized back to Cu^+^, which is in agreement with the electrochemical results that show that only half of the CuS capacity can be extracted after the charge process. The larger broadness of Cu 2p_3/2_ peak for the charge state in comparison with pristine and discharge states indicates on the different chemical environment of Cu^+^ sites on the surface and might be related to the formation of SEI on the CuS electrodes at high voltages. The latter could be a reason of limited oxidation reaction of the Cu upon charge. As shown in Fig. 3c (XPS) and Fig. 3d (EDX), the Mg peak appears after discharge, and is half the intensity after the following charge. The oxidation state of sulfur is S;^2−^ however, it does not change while cycling (Fig. 3b), indicating that Cu is the only redox-active element in the magnesiation/de-magnesiation of CuS NPs. Following the above discussion, the initial discharge process for CuS NPs can be described according to the following equation:

1 $$CuS+M{g}^{2+}+2{e}^{-}\to Cu+MgS$$

From the cycling, the charge/discharge reactions can be presented as:

2 $$2Cu+MgS\leftrightarrow C{u}_{2}S+M{g}^{2+}+2{e}^{-}$$

We note that the proposed electrochemical reactions are in line with the CuS magnesiation mechanism reported by Nazar *et al*.^44^, which includes a two-step reduction of CuS, eventually forming MgS and Cu. As follows from EDX results (Fig. 3d), the part of MgS being formed during first discharge is trapped and can not be recovered upon the charge.

The crystallographic phase change in the CuS NPs after discharge were identified via *ex situ* XRD analysis (Figure S5). The decrease in intensity from the CuS diffraction peaks after discharge indicated that magnesiation of CuS NPs occurred with continuous amorphization of the material. We suspect that phase transitions within the amorphized electrode can lead to lower mechanical stress during cycling, compared with that of crystalline NPs, which may explain the high cycling stability that was observed for the CuS NPs. The superior performance of CuS NPs can be also attributed to the amorphous MgS (irreversibly formed on the first cycle) acting as a matrix, buffering volume changes in CuS electrode. In addition, the amorphization of CuS NPs during cycling might facilitate magnesiation/de-magnesiation reactions, thereby leading to higher use of the capacity, as indicated by the increasing capacity values during initial cycling (Fig. 2b).

**Figure 4:**
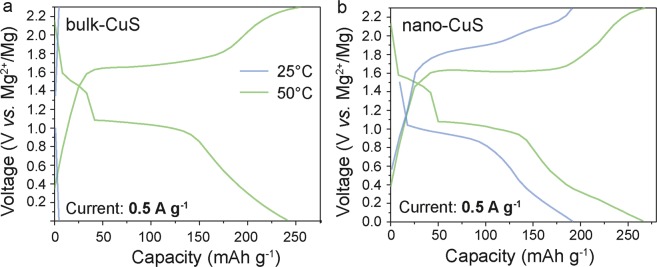
Typical galvanostatic charge-discharge curves of CuS NPs (**a**) and microcrystalline CuS (**b**) measured at a current density of 0.5 A g^−1^ and at a temperature of 25 and 50 °C, respectively.

Figure 4a,b compare the voltage profiles of CuS NPs with its bulk counterpart, as measured at a current density of 0.5 A g^−1^. To compare the favorably intrinsic electrochemical behavior of both nano and bulk CuS, the electrode formulation (amounts of binder and conductive additive), electrode thickness and electrolyte were fixed in our experiments. As follows from Fig. 4, nano-CuS showed superior electrochemical performance over bulk CuS (Figure S6). At room temperature, bulk CuS did not show electrochemical activity, which was only improved at an elevated temperature of 50 °C. In contrast, the electrochemical performance of nano-CuS only slightly improved at higher temperature. Similar electrochemical cycling behaviors were obtained with Cu_2_S NPs (Figure S7). Namely, a stable capacity of 200 mAh g^−1^ was retained over at least 50 cycles, clearly outperforming bulk Cu_2_S (Figure S6, S8), which showed very low electrochemical activity at room temperature. Apparently, decreasing the size of the copper sulfide particles significantly enhances the kinetics of magnesiation/de-magnesiation which is associated with the increase of the active area of the material/electrolyte interface. As a result, the use of CuS in the form of NPs gave drastically enhanced activity in comparison with microcrystalline bulk particles.

## Conclusions

In summary, we have reported CuS NPs as new cathode materials for Mg-ion storage. These CuS NPs exhibit high capacities of approximately 300 mAh g^−1^ at a current density of 100 mA g^−1^ (~1 C) and high cyclic stability over 200 cycles. The magnesiation/demagnesiation mechanism was studied by *ex situ* XPS and EDX measurements and suggests the reduction/oxidation of copper during the discharging/charging of CuS NPs, wherein sulfur in the form of S^2−^ does not change during cycling. A side-by-side comparison of nano and bulk CuS showed that the electrochemical performance of bulk CuS is only comparable with that of nano CuS at an elevated temperature of 50 °C, and its performance was negligible at room temperature. These results highlight that nanostructuring is an effective approach to overcome kinetic limitations of conversion-type copper (II) sulfide cathodes that are observed in the bulk system.

## Methods

### Chemicals

Copper (I) chloride (99.99%, STREM), Oleylamine (Oam, 95%, STREM), Oleic acid (OA, 90%, Aldrich), Octadecene (ODE, 90%, Sigma-Aldrich), Copper (I) acetate (ABCR), Copper (II) acetylacetonate (97%, Sigma-Aldrich), TOPO (Strem, 99%), chlorobenzene (anhydrous, 99.8%, Sigma-Aldrich), Sulfur (99.998%, Sigma-Aldrich), 1-dodecanethiol (98%, Sigma Aldrich), Toluene (99.9%, Sigma-Aldrich), Ethanol (Fluka), Hydrazine (Gerling Holz+Co), Acetonitrile (ACN, max. 0.005% H_2_O, Merck), Copper (II) sulfide (CuS, 99.5%, STREM), Copper (I) sulfide (Cu_2_S, 99.5%, Alfa Aesar), Magnesium-bis(hexamethyldisilazide (Mg(HMDS)_2_, 97%, Sigma-Aldrich), Magnesium bis(trifluoromethanesulfonyl)imide (Mg(TFSI)_2_, 99.5%, Solvionic), Magnesium chloride (MgCl_2_, 99.9%, Alfa Aesar), Tetraglyme (99%, ABCR).

### Battery components

Carbon black (Super C65, provided by TIMCAL), a glass-fiber separator (EUJ-grade, Hollingsworth & Vose Company Ltd., United Kingdom), Poly(vinylidene fluoride) (PVDF, average Mw ~534 g/mol, Sigma Aldrich), N-Methyl-2-pyrrolidone (NMP, 99%, Sigma Aldrich).

### Synthesis of CuS NPs

Copper (II) sulfide nanocrystals (NPs) were synthesized using a previously reported method^49^. Copper (I) chloride (49 mg), oleylamine (2 ml), oleic acid (0.21 ml) and octadecene (8 ml) were mixed in a three-neck flask and degassed under vacuum for 20 min at 100 °C and then 20 min at 130 °C. Then, the mixture was heated to 180 °C under Ar flow, and 2 ml of a 1 M sulfur solution in oleylamine was injected into the flask. The solution was allowed to react for 10 min at 180 °C and then cooled down to room temperature. NPs were purified with a chloroform/acetone mixture twice and then stored in chloroform (3 ml).

### Synthesis of Cu_2_S NPs

Copper (I) sulfide NPs were synthesized using a previously reported method^53^. Copper (I) acetate (48 mg), TOPO (1 g) and octadecene (30 ml) were mixed in a three-neck flask, degassed under vacuum for 30 min at 100 °C and then heated to 160 °C. At 160 °C, a quick injection of 1-dodecanethiol (1 ml) was performed, and the solution was again heated to 210 °C. The solution was allowed to react for 80 min at 210 °C, and then cooled down naturally to room temperature. NPs were purified with a chloroform/ethanol mixture twice, and finally stored in chloroform (3 ml).

### Ligand removal

Ligands were removed from the surface of the NPs by treatment with hydrazine. NPs were stirred for 2 hours in a solution of hydrazine (0.8 ml) in anhydrous acetonitrile (25 ml). Afterwards, the material was washed three times with acetonitrile and dried under vacuum at room temperature.

### Preparation of Mg/CuS or Mg/Cu_2_S cells and electrochemical testing

CuS or Cu_2_S electrodes were prepared by ball-milling the respective CuS or Cu_2_S NPs after ligand removal or bulk material (64 wt%) with carbon black (21 wt%) and PVDF binder (15 wt%) in NMP for 1 h and casting the obtained slurry onto a tungsten current collector. The current collectors were then dried for 12 h at 80 °C. Coin-type cells were assembled in a glovebox using a one layer glass fiber separator. Polished Mg metal served as both the reference and counter electrode. A solution of Mg(HMDS)_2_/AlCl_3_/MgCl_2_ in tetraglyme was used as the Mg electrolyte^51,52^. Assembled cells were cycled using a MPG2 multi-channel workstation (Bio-Logic). The obtained capacities were normalized to the mass of the CuS or Cu_2_S active materials.

### Materials Characterization

TEM images were obtained using a JEOL JEM-2200FS microscope operating at 200 kV. Carbon-coated TEM grids from Ted-Pella were used as substrates. For element mapping using HAADF-STEM combined with EDX, a beam current of 6 nA (no difference when measured with 1 or 3 nA) and detection with a SuperEDX system (4 detectors) were used. The measurements were performed on an FEI Titan Themis microscope operated at 300 kV with a probe semiconvergence angle of 18 mrad (beam current 70 pA). EDX spectroscopy maps were collected on an FEI Talos F200X microscope operated at 200 kV. SEM images were measured using a Hitachi 3030Plus tabletop electron microscope. Powder XRD was measured on a STOE STADI P diffractometer (Cu-Kα_1_ irradiation, λ = 1.540598 Å). XPS measurements were carried out in normal emission using a monochromatized Al Kα X-ray radiation source and a Scienta R3000 display analyzer. For the quantification of the near-surface composition, core levels of S2s, Cu2p and Mg2s were fitted with Voigt-Lorenthian curves using XPSmania (implemented in Igor Pro) which was kindly provided by the ALOISA beamline staff of the ELETTRA synchrotron facility. The energy dependency of the mean free path was included in the photoionization cross sections^54^ resulting in the following values (see Table S1).

## Supplementary information

Supporting Information

## References

[1] Yoo HD (2013). Mg Rechargeable Batteries: an On-going Challenge. Energy Environ. Sci..

[2] Davidson R (2019). Formation of magnesium dendrites during electrodeposition. ACS Energy Lett..

[3] Bucur CB, Gregory T, Oliver AG, Muldoon J (2015). Confession of a Magnesium Battery. J. Phys. Chem. Lett.

[4] Saha P (2014). Rechargeable Magnesium Battery: Current Status and Key Challenges for the Future. Prog. Mater. Sci..

[5] Muldoon J, Bucur CB, Gregory T (2014). Quest for nonaqueous multivalent secondary batteries: Magnesium and beyond. Chem. Rev..

[6] Doe RE (2014). Novel, Electrolyte Solutions Comprising Fully Inorganic Salts with High Anodic Stability for Rechargeable Magnesium Batteries. Chem. Commun..

[7] Zhang Z (2017). Novel Design Concepts of Efficient Mg‐Ion Electrolytes toward High‐Performance Magnesium–Selenium and Magnesium–Sulfur Batteries. Adv. Energy Mater..

[8] Tutusaus O (2015). An Efficient Halogen‐Free Electrolyte for Use in Rechargeable Magnesium Batteries. Angew. Chem. Int. Ed..

[9] Zhao‐Karger Z (2015). Performance Improvement of Magnesium Sulfur Batteries with Modified Non‐Nucleophilic Electrolytes. Adv. Energy Mater..

[10] Muldoon J, Bucur CB, Gregory T (2017). Fervent Hype behind Magnesium Batteries: An Open Call to Synthetic Chemists—Electrolytes and Cathodes Needed. Angew. Chem. Int. Ed..

[11] Levi E, Levi MD, Chasid O, Aurbach D (2009). A Review on the Problems of the Solid State Ions Diffusion in Cathodes for Rechargeable Mg Batteries. J. Electroceram..

[12] Wan LF, Perdue BR, Apblett CA, Prendergast D (2015). Mg Desolvation and Intercalation Mechanism at the Mo_6_S_8_ Chevrel Phase Surface. Chem. Mater..

[13] Aurbach D (2007). Progress in Rechargeable Magnesium Battery Technology. Adv. Mater..

[14] Mohtadi R, Fuminori Mizuno F (2014). Magnesium Batteries: Current State of the Art, Issues and Future Perspectives. Beilstein J. Nanotechnol..

[15] Huie MM, Bock DC, Takeuchi ES, Marschilok AC, Takeuchi KJ (2015). Cathode Materials for Magnesium and Magnesium-ion Based Batteries. Coord. Chem. Rev..

[16] Bonnick P, Sun X, Lau K-C, Liao C, Nazar LF (2017). Monovalent versus Divalent Cation Diffusion in Thiospinel Ti_2_S_4_. J. Phys. Chem. Lett.

[17] Thole F, Wan LF, Prendergast D (2015). Re-examining the Chevrel Phase Mo_6_S_8_ Cathode for Mg Intercalation from an Electronic Structure Perspective. Phys. Chem. Chem. Phys..

[18] Liu M (2016). Evaluation of Sulfur Spinel Compounds for Multivalent Battery Cathode Applications. Energy Environ. Sci..

[19] Sun X, Bonnick P, Nazar LF (2016). Layered TiS_2_ Positive Electrode for Mg Batteries. ACS Energy Lett..

[20] Liu Y, Li Y, Kang H, Jin T, Jiao L (2016). Design, Synthesis, and Energy-related Applications of Metal Sulfides. Mater. Horiz..

[21] Aurbach D (2000). Prototype Systems for Rechargeable Magnesium Batteries. Nature.

[22] Ji‐Lei S (2018). High‐Capacity Cathode Material with High Voltage for Li‐Ion Batteries. Adv. Mater..

[23] Nitta N, Wu F, Lee JT, Yushin G (2015). Li-ion Battery Materials: Present and Future. Mater. Today.

[24] Oszajca MF (2015). Colloidal BiF_3_ nanocrystals: a bottom-up approach to conversion-type Li-ion cathodes. Nanoscale.

[25] Guntlin CP (2017). Nanocrystalline FeF_3_ and MF_2_ (M=Fe, Co, and Mn) from metal trifluoroacetates and their Li(Na)-ion storage properties. J. Mater. Chem. A.

[26] Kravchyk KV, Zünd T, Wörle M, Kovalenko MV, Bodnarchuk MI (2018). NaFeF_3_ nanoplates as low-cost sodium and lithium cathode materials for stationary energy storage. Chem. Mater..

[27] Tao, Z.-L., Xu, L.-N., Gou, X.-L., Chen, J. & Yuan, H.-T. TiS_2_ Nanotubes as the Cathode Materials of Mg-ion Batteries. *Chem. Commun*. **0**, 2080–2081 (2004). 10.1039/b403855j 15367984

[28] Yanliang L (2011). Rechargeable Mg Batteries with Graphene‐like MoS_2_ Cathode and Ultrasmall Mg Nanoparticle Anode. Adv. Mater..

[29] Kim R-H (2014). Highly reduced VOx nanotube cathode materials with ultra-high capacity for magnesium ion batteries. J. Mater. Chem. A.

[30] Tepavcevic S (2015). Nanostructured Layered Cathode for Rechargeable Mg-Ion Batteries. ACS Nano.

[31] Amatucci GG (2001). Investigation of Yttrium and Polyvalent Ion Intercalation into Nanocrystalline Vanadium Oxide. J. Electrochem. Soc..

[32] Sian TS, Reddy GB (2004). Infrared and Electrochemical Studies on Mg Intercalated a-MoO_3_ Thin Films. Solid State Ionics.

[33] Liang Y (2015). Interlayer-Expanded Molybdenum Disulfide Nanocomposites for Electrochemical Magnesium Storage. Nano Lett..

[34] He M, Kravchyk K, Walter M, Kovalenko MV (2014). Monodisperse Antimony Nanocrystals for High-Rate Li-ion and Na-ion Battery Anodes: Nano versus Bulk. Nano Lett..

[35] Oszajca MF, Bodnarchuk MI, Kovalenko MV (2014). Precisely Engineered Colloidal Nanoparticles and Nanocrystals for Li-Ion and Na-Ion Batteries: Model Systems or Practical Solutions?. Chem. Mater..

[36] Liu Junfeng, Wang Shutao, Kravchyk Kostiantyn, Ibáñez Maria, Krumeich Frank, Widmer Roland, Nasiou Déspina, Meyns Michaela, Llorca Jordi, Arbiol Jordi, Kovalenko Maksym V., Cabot Andreu (2018). SnP nanocrystals as anode materials for Na-ion batteries. Journal of Materials Chemistry A.

[37] Wang S (2018). Monodisperse CoSn_2_ and FeSn_2_ Nanocrystals as High-performance Anode Materials for Lithium-ion Batteries. Nanoscale.

[38] Walter M, Bodnarchuk MI, Kravchyk KV, Kovalenko MV (2015). Evaluation of Metal Phosphide Nanocrystals as Anode Materials for Na-ion Batteries. Chimia.

[39] Jache B, Mogwitz B, Klein F, Adelhelm P (2014). Copper Sulfides for Rechargeable Lithium Batteries: Linking Cycling Stability to Electrolyte Composition. J. Power Sources.

[40] Cai R (2012). Synthesis of CuxS/Cu Nanotubes and Their Lithium Storage Properties. J. Phys. Chem. C.

[41] Zhao L (2012). Bubble Template Synthesis of Copper Sulfide Hollow Spheres and Their Applications in Lithium ion Battery. Mater. Lett..

[42] Wang Y, Zhang X, Chen P, Liao H, Cheng S (2012). *In situ* Preparation of CuS Cathode with Unique Stability and High Rate Performance for Lithium Ion Batteries. Electrochim. Acta.

[43] Tashiro Y, Taniguchi K, Miyasaka H (2017). The Effect of Anion-sublattice Structure on the Displacement Reaction in Copper Sulfide Cathodes of Rechargeable Magnesium Batteries. Chem. Lett..

[44] Duffort V, Sun X, Nazar LF (2016). Screening for Positive Electrodes for Magnesium Batteries: a Protocol for Studies at Elevated Temperatures. Chem. Commun..

[45] Li T (2018). A High-performance Hybrid Mg^2+^/Li^+^ Battery Based on Hierarchical Copper Sulfide Microflowers Conversion Cathode. Electrochim. Acta.

[46] Xiong F (2018). Magnesium storage performance and mechanism of CuS cathode. Nano Energy.

[47] Chung JS, Sohn HJ (2002). Electrochemical Behaviors of CuS as a Cathode Material for Lithium Secondary Batteries. J. Power Sources.

[48] Wu M (2018). Copper sulfide nanoparticles as high-performance cathode materials for magnesium secondary batteries. Nanoscale.

[49] Xie Y (2013). Metallic-like Stoichiometric Copper Sulfide Nanocrystals: Phase- and Shape-Selective Synthesis, Near-Infrared Surface Plasmon Resonance Properties, and Their Modeling. ACS Nano.

[50] Talapin DV, Murray CB (2005). PbSe Nanocrystal Solids for n- and p-Channel Thin Film Field-Effect Transistors. Science.

[51] Zhao-Karger Z, Zhao X, Fuhr O, Fichtner M (2013). Bisamide Based Non-nucleophilic Electrolytes for Rechargeable Magnesium Batteries. RSC Adv..

[52] Vinayan BP (2016). Performance Study of Magnesium-sulfur Battery Using a Graphene Based Sulfur Composite Cathode Electrode and a Non-nucleophilic Mg Electrolyte. Nanoscale.

[53] Wang Y (2010). One-Pot Synthesis and Optical Property of Copper(I) Sulfide Nanodisks. Inorg. Chem..

[54] Yeh JJ, Lindau I (1985). Atomic subshell photoionization cross sections and asymmetry parameters: 1 ≤ Z ≤ 103. At. Data Nucl. Data Tables.

