# Supplementary material for: Copper sulfide nanoparticles as high-performance cathode materials for Mg-ion batteries
Source: Sci Rep. 2019 May 29;9:7988. doi: 10.1038/s41598-019-43639-z (PMC6541626; doi:10.1038/s41598-019-43639-z)
Supplement: Supplementary file 1 — Supporting Information [file 41598_2019_43639_MOESM1_ESM.docx]

*Supporting Information for*

Copper sulfide nanoparticles as high-performance cathode materials for Mg-ion batteries

Kostiantyn V. Kravchyk,^1,2^ Roland Widmer, ^3^ Rolf Erni, ^4^ Romain J.-C. Dubey, ^1,2^ Frank Krumeich,^2^ Maksym V. Kovalenko,^*,1,2^ and Maryna I. Bodnarchuk ^*,2^

^1^ Laboratory of Inorganic Chemistry, Department of Chemistry and Applied Biosciences, ETH Zürich, Vladimir-Prelog-Weg 1, CH-8093 Zürich, Switzerland

^2^ Laboratory for Thin Films and Photovoltaics, Empa – Swiss Federal Laboratories for Materials Science and Technology, Überlandstrasse 129, CH-8600 Dübendorf, Switzerland

^3^ Nanotech@surfaces Laboratory, Empa – Swiss Federal Laboratories for Materials Science and Technology, Überlandstrasse 129, CH-8600 Dübendorf, Switzerland

^4^ Electron Microscopy Center, Empa – Swiss Federal Laboratories for Materials Science and Technology, Überlandstrasse 129, CH-8600 Dübendorf, Switzerland

Corresponding Authors:

*E-mail: [maryna.bodnarchuk@empa.ch](file:///C:\Users\kro\Documents\NaFeF3%20paper\ASC%20Energy%20Letters\maryna.bodnarchuk@empa.ch) and [mvkovalenko@ethz.ch](mailto:mvkovalenko@ethz.ch)


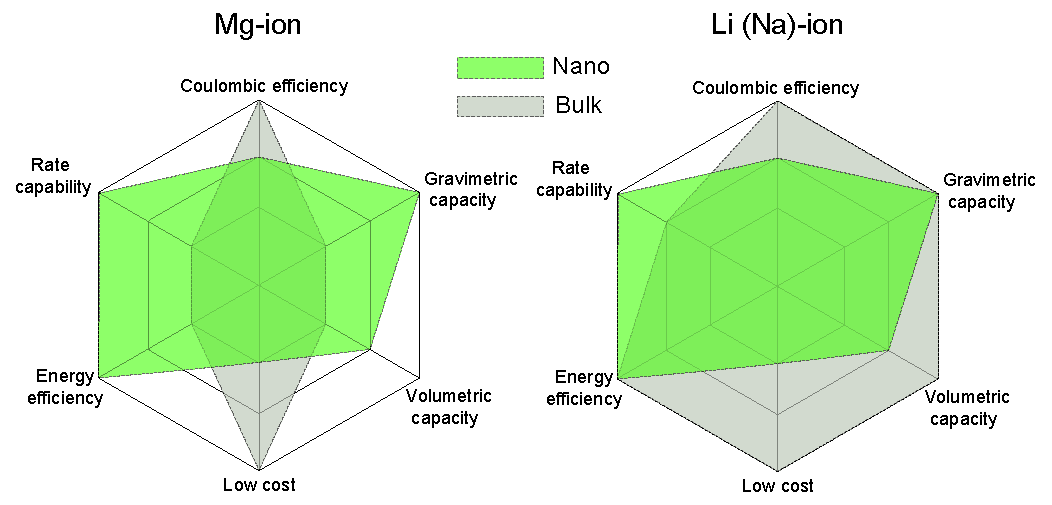


**Figure S1.** Spider chart of the advantages and disadvantages of nano- and bulk cathode materials for Mg-ion and Li-ion batteries. The centers and edges of the hexagons represent the lowest and highest values, respectively, of the coulombic efficiency, gravimetric capacity, volumetric capacity, low cost, energy efficiency and rate capability.

**
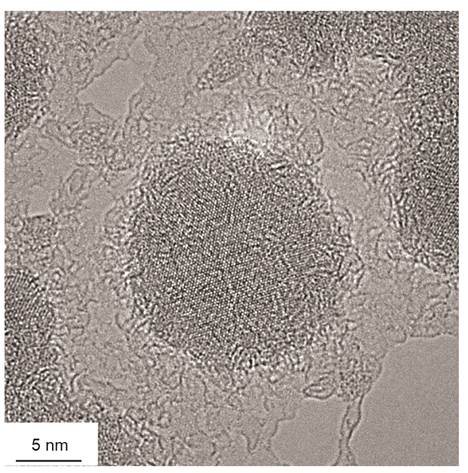
**

**Figure S2.** HRTEM image of CuS NPs.


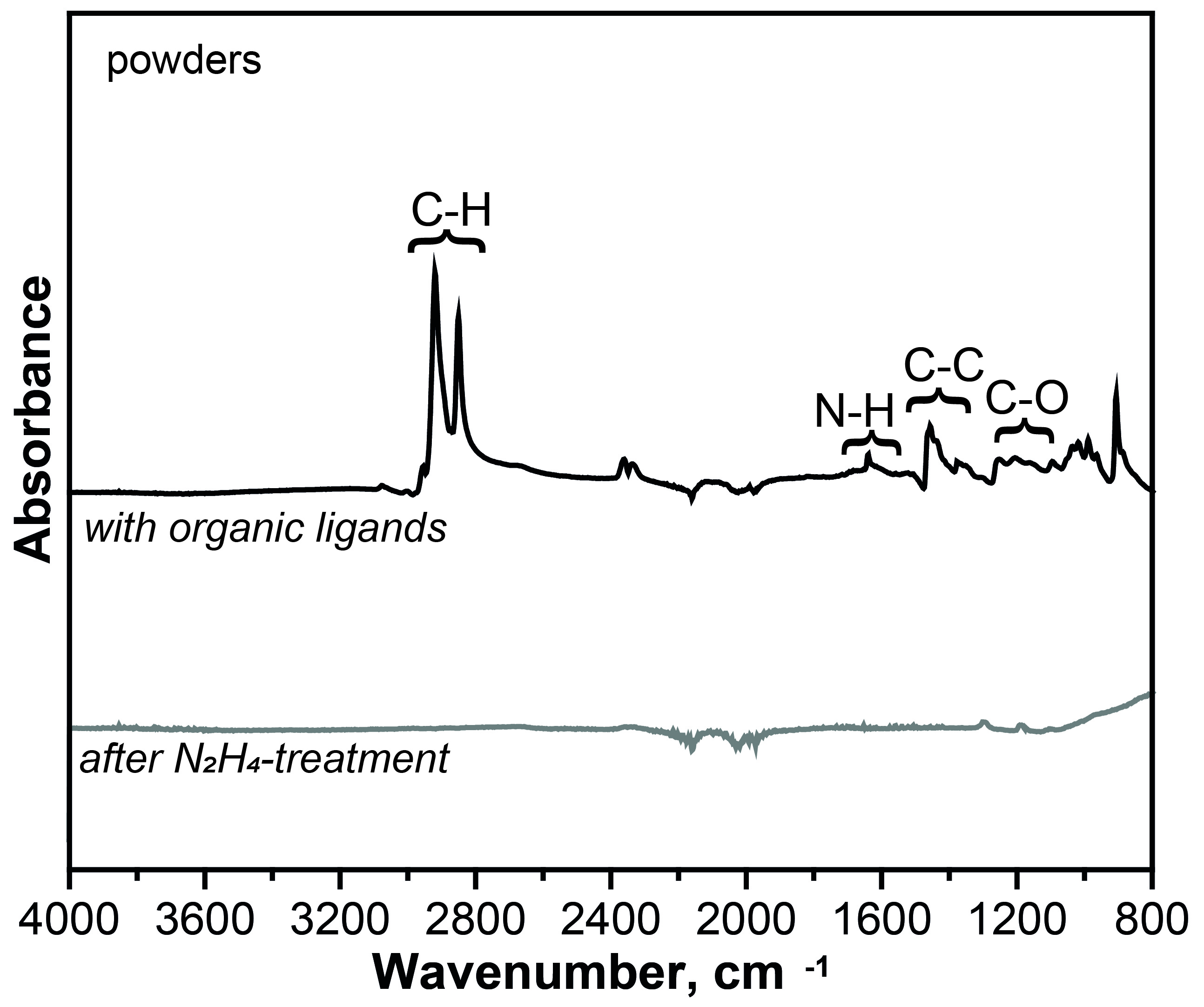


**Figure S3**. FTIR spectra of CuS NPs before (after the synthesis) and after ligand removal. The absence of C-H, N-H, C-C and C-O bonds after ligand removal indicated that all organic ligands were removed by hydrazine treatment.


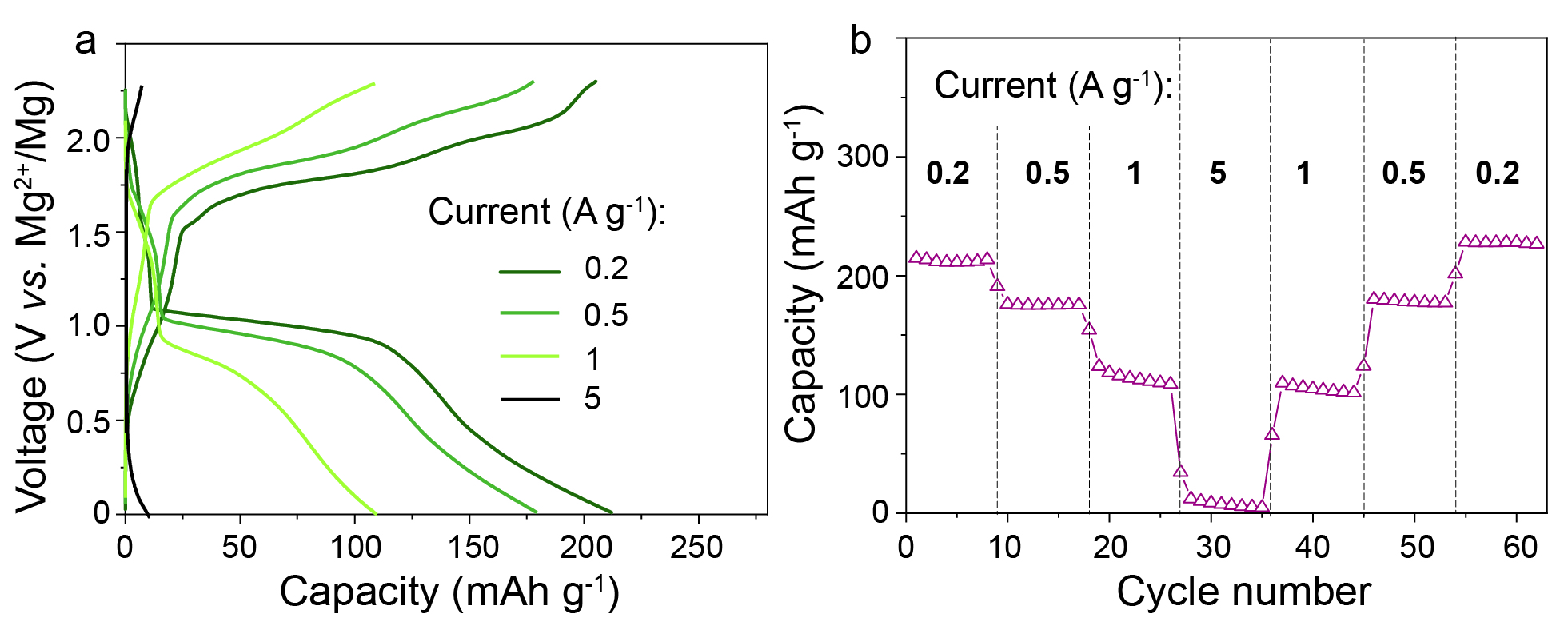


**Figure S4.** (a) Galvanostatic charge-discharge curves and (b) cyclic stability of CuS NPs measured at current densities of 0.2, 0.5, 1 and 5 A g^-1^.

**
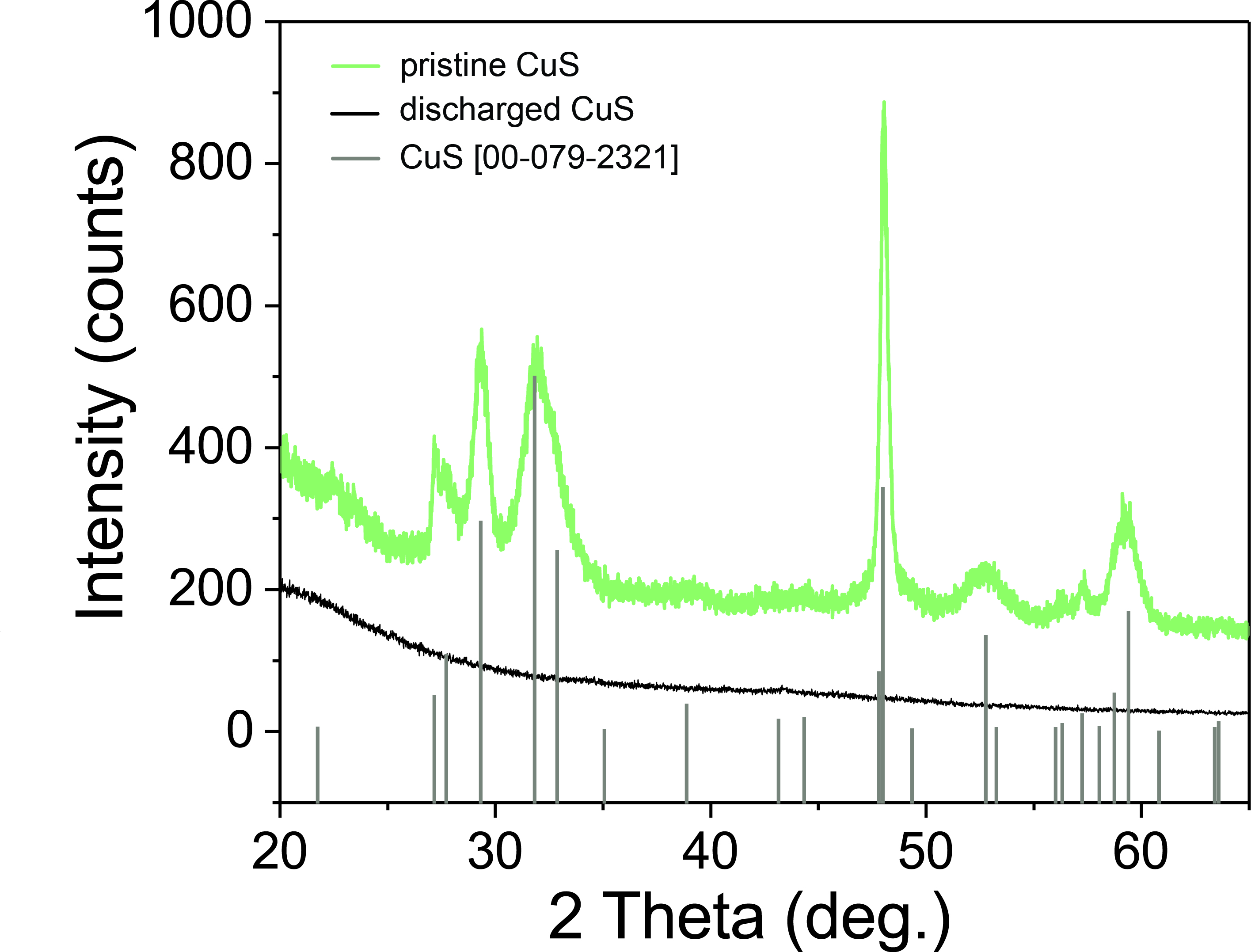
**

**Figure S5.** *Ex situ* XRD pattern of CuS NPs discharged at a current density of 0.1 A g^-1^; XRD pattern of pristine CuS NPs is given for comparison.


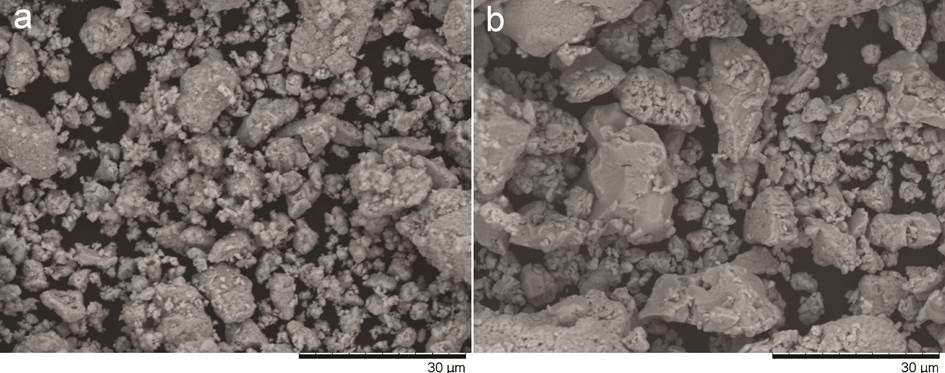


**Figure S6.** SEM images of bulk CuS (a) and Cu_2_S (b) particles.


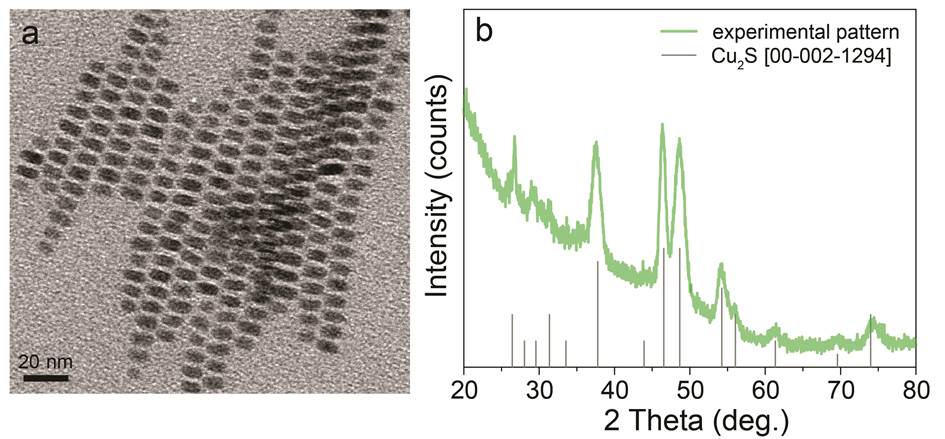


**Figure S7.** TEM image (a) and XRD pattern (B) of Cu_2_S NPs.


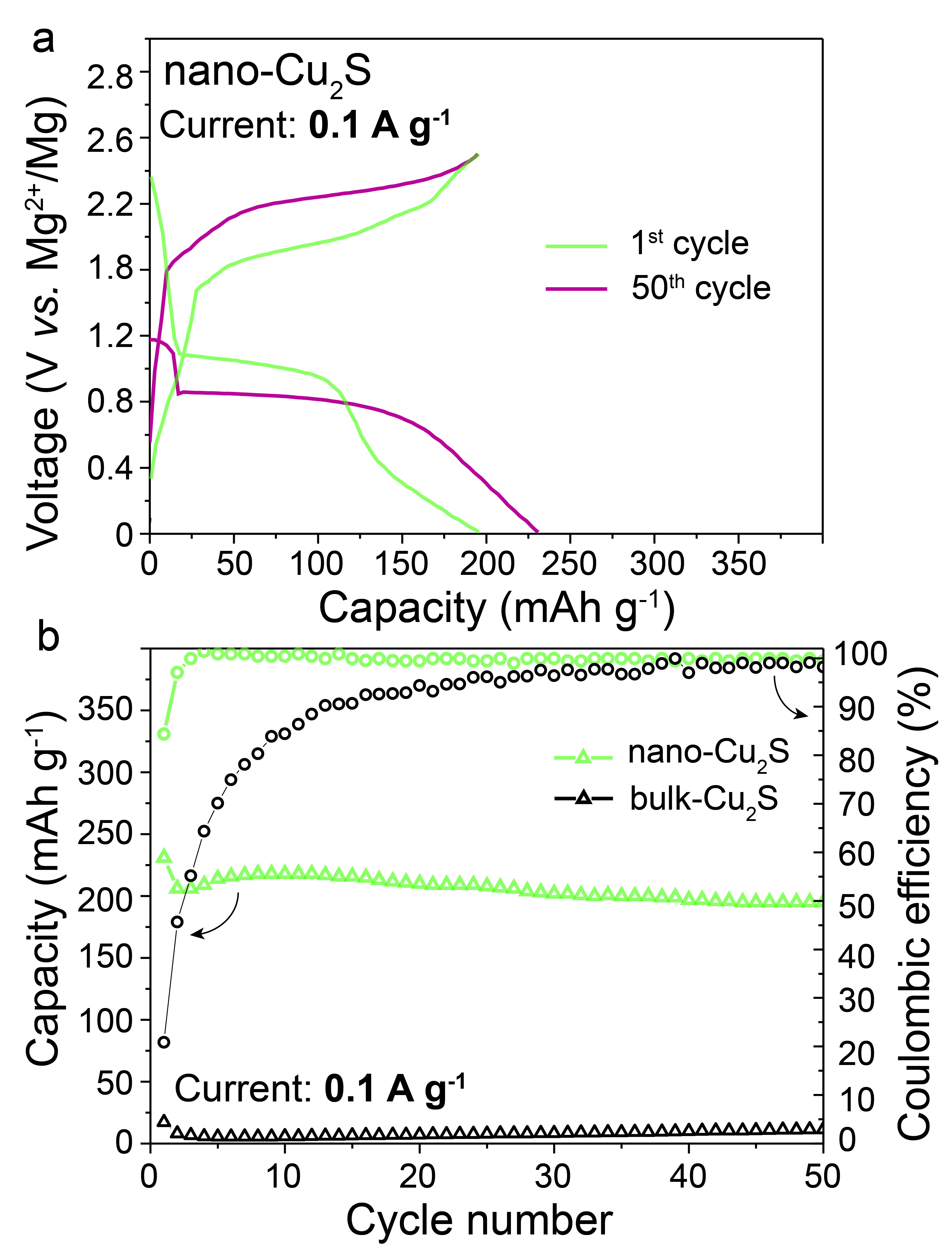


**Figure S8.** Electrochemical performance of Cu_2_S NPs. (a) Galvanostatic charge-discharge curves during 1^st^ and 50^th^ cycle at a current density of 0.1 A g^-1^; (b) Cyclic stability measured at a current density of 0.1 A g^-1^.

**Table S1.** Atomic ratios of S, Cu and Mg for pristine, discharged and charged CuS NPs derived from corresponding XPS spectra (Figure 3, main text).

|  | S | Cu | Mg |
| --- | --- | --- | --- |
| Pristine | 1 | 0.96 | - |
| Discharged | 1 | 1.08 | 2.73 |
| Charged | 1 | 1.64 | 1.38 |
